# Identification of candidate miRNA biomarkers from miRNA regulatory network with application to prostate cancer

**DOI:** 10.1186/1479-5876-12-66

**Published:** 2014-03-11

**Authors:** Wenyu Zhang, Jin Zang, Xinhua Jing, Zhandong Sun, Wenying Yan, Dongrong Yang, Feng Guo, Bairong Shen

**Affiliations:** 1 Center for Systems Biology, Soochow University, Suzhou 215006, China; 2 Department of Urology, the First Affiliated Hospital of Soochow University, Suzhou 215006, China; 3 Department of Urology, the Second Affiliated Hospital of Soochow University, Suzhou 215004, China; 4 Central lab, the First Affiliated Hospital of Soochow University, Suzhou 215006, China

**Keywords:** miRNA biomarker, Gene expression, miRNA regulatory network, Prostate cancer

## Abstract

**Background:**

MicroRNAs (miRNAs) are a class of non-coding regulatory RNAs approximately 22 nucleotides in length that play a role in a wide range of biological processes. Abnormal miRNA function has been implicated in various human cancers including prostate cancer (PCa). Altered miRNA expression may serve as a biomarker for cancer diagnosis and treatment. However, limited data are available on the role of cancer-specific miRNAs. Integrative computational bioinformatics approaches are effective for the detection of potential outlier miRNAs in cancer.

**Methods:**

The human miRNA-mRNA target network was reconstructed by integrating multiple miRNA-mRNA interaction datasets. Paired miRNA and mRNA expression profiling data in PCa versus benign prostate tissue samples were used as another source of information. These datasets were analyzed with an integrated bioinformatics framework to identify potential PCa miRNA signatures. In vitro q-PCR experiments and further systematic analysis were used to validate these prediction results.

**Results:**

Using this bioinformatics framework, we identified 39 miRNAs as potential PCa miRNA signatures. Among these miRNAs, 20 had previously been identified as PCa aberrant miRNAs by low-throughput methods, and 16 were shown to be deregulated in other cancers. In vitro q-PCR experiments verified the accuracy of these predictions. miR-648 was identified as a novel candidate PCa miRNA biomarker. Further functional and pathway enrichment analysis confirmed the association of the identified miRNAs with PCa progression.

**Conclusions:**

Our analysis revealed the scale-free features of the human miRNA-mRNA interaction network and showed the distinctive topological features of existing cancer miRNA biomarkers from previously published studies. A novel cancer miRNA biomarker prediction framework was designed based on these observations and applied to prostate cancer study. This method could be applied for miRNA biomarker prediction in other cancers.

## Background

MicroRNAs (miRNAs) are a class of small non-coding RNAs of approximately 22 nucleotides in length with the potential to regulate human genes through translation inhibition or mRNA cleavage [1]. Recent studies have shown that miRNAs are involved in a wide variety of biological processes such as cell proliferation [2], development [3], and apoptosis [4]. Abnormal expression of miRNAs has been implicated in various human cancers and may constitute a potential signature for cancer diagnosis [5-7]. However, limited data on cancer related miRNAs are available, and their regulatory mechanisms remain largely unknown.

Extensive research efforts have focused on the identification of potential cancer miRNA biomarkers [6-10]. The preliminary detection of differentially expressed (DE) miRNAs from large-scale miRNA expression profiling data and low-throughput experimental validation for selected outlier miRNAs are the routine methods used in these studies. As the activities of outlier miRNAs are at least partially reflected in the aberrant expression of their target genes [11], systematic computational approaches that integrate miRNA regulatory data and gene expression profiling data were shown to be more effective to infer potential outlier miRNA activities in cancers [11,12].

MiRNAs are known to function in a multiple-to-multiple relationship with their target genes, and a concept referred to as miRNA regulation module was proposed based on this theory [13]. This idea was further explored in cancer studies, and attempts have been made to identify candidate abnormal miRNAs or miRNA regulatory modules in cancer [12,14-17]. The assumption that abnormal miRNAs associated with cancer show increased functional synergism because of their co-regulatory effects on the same genes [18] was the underlying foundation of these computational approaches.

**Figure 1:**
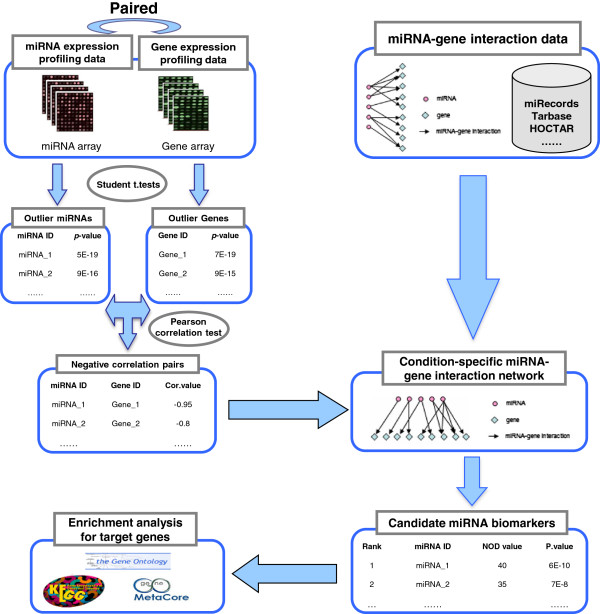
Schematic workflow for the identification of potential cancer miRNA biomarkers.

In contrast to these analyses, the miRNA regulatory network was shown to follow power-law distribution in our study. This implies that more miRNAs tend to have fewer target genes and fewer genes have more miRNA regulators. Based on this analysis, we defined a novel out degree (NOD) measure to characterize the independent regulatory power of individual miRNAs, i.e., the number of genes uniquely regulated by one specific miRNA. Our analysis indicated that miRNAs with larger NOD values are statistically more likely to be candidate cancer biomarkers, which implies that abnormal miRNAs in cancer generally have independent regulatory power. Based on this observation, we proposed a novel pipeline to infer candidate cancer miRNA biomarkers, and applied this method to prostate cancer (PCa). The schematic workflow of our pipeline is shown in Figure 1. Paired miRNA and mRNA expression profiling data for PCa, and reliable miRNA-mRNA interaction data were integrated to generate a PCa conditional miRNA regulatory network, and then candidate PCa miRNA biomarkers in the above conditional network were prioritized according to their independent regulatory power. PubMed citation analysis of the known PCa abnormal miRNAs and in vitro q-PCR technology were used to verify the accuracy of our prediction results. Finally, systematic methods were applied to explore the relationship between PCa and the unique target genes of candidate PCa miRNA biomarkers. The results indicated that our method can detect potential miRNA-mRNA target relationships in specific cancers and can be applied for the identification of miRNA biomarkers in cancer.

## Methods

### Dataset collection

Expression profiles (GSE34933 from NCBI GEO) for PCa and benign prostate tissue (BPH) samples generated by Zhong and colleagues [19,20] were used. Eight available paired miRNA and mRNA expression profiles (each containing 4 samples for PCa and BPH) were selected for further analysis. Information on these profiles is provided in Additional file 1. Normalized miRNA and mRNA data were downloaded directly. For mRNA expression data, the average probe intensity was calculated and used as the gene expression level for genes with multiple probes. Finally, the profiles included information on the expression of 851 miRNAs and 19595 genes.

Another dataset used in this study was the miRNA-mRNA network. This dataset consisted of a combination of experimentally validated targeting data and computational prediction data. The experimentally validated data included information from miRecords [21], TarBase [22], miR2Disease [23], and miRTarBase [24], while the computational prediction data consisted of miRNA-mRNA target pairs residing in no fewer than 2 datasets from HOCTAR [25], ExprTargetDB [26], and starBase [27]. In total, there were 32739 regulatory pairs among 641 miRNAs and 7706 target genes.

### Prostate cancer miRNA biomarker identification

We developed a novel approach to identify candidate miRNA biomarkers for PCa. The schematic workflow of our pipeline is described in Figure 1. Paired miRNA and gene expression, and miRNA-mRNA networks were integrated to predict outlier miRNAs associated with PCa progression. This procedure consisted of four separate stages. First, differentially expressed miRNAs and genes between PCa and BPH samples were detected using the two-sample t-test. Second, Pearson’s correlation was used to detect negative correlations between the expression profiles of outlier miRNAs and outlier genes. In the third step, the intersection data of the negative correlations and miRNA-mRNA binding pairs were retrieved to identify miRNA regulatory networks related to PCa progression. In the fourth and final step, a new index designated as novel out-degree (NOD) was defined to measure the independent regulatory power of an individual miRNA, and used to prioritize novel PCa miRNA biomarkers.

### Step 1: Detection of differentially expressed miRNAs and genes associated with prostate cancer

The detection of cancer-specific abnormal changes in miRNA and gene expression is the aim of cancer studies [28-31]. Here, we used two-sample t-tests to identify differently expressed miRNAs and genes associated with PCa progression on the basis of their expression profiles. The top 30% miRNAs (or genes) ranked by their statistical significance (*p*-value) were retrieved for further analysis. As a result, 256 miRNAs and 5878 genes were considered as candidate PCa outliers.

The threshold for the expression of outlier miRNAs and outlier genes is often arguable. A less stringent cut-off (top 40%) and a stricter cut-off (top 20%) were tested for candidate miRNA biomarker prediction. Details of the comparison between these predictions are listed in Additional file 2. The data indicated that the prediction results were highly conserved and only the number of candidate miRNAs changed with the different thresholds. Therefore, we adopted a moderate threshold (top 30%) in the present study.

### Step 2: Acquisition of inverse correlation pairs

One major function of miRNAs is the cleavage of transcripts of its target genes at the post-transcriptional level. Thus, the inverse correlation of expression profiles should be one prerequisite for miRNAs and candidate targets. In the present study, the Pearson’s correlation method was used to detect negative correlations between outlier miRNAs and outlier genes. The cut-off for the correlation coefficient was roughly chosen to be -0.6, as it has been used as a threshold in several correlation studies [32,33].

### Step 3: Constructing a prostate cancer miRNA-mRNA binding network

According to the above miRNA-mRNA binding data from experimental validation and computational prediction databases, we identified possible human miRNA-mRNA target pairs. We further filtered these target pairs with the collected information on miRNA-mRNA negative correlations to generate a PCa miRNA regulatory network. As a result, the miRNA-mRNA target sub-network consisted of 136 miRNAs and 551 target genes.

### Step 4: Prioritizing candidate prostate cancer miRNA biomarkers

Generally, we face two main challenges for the prediction of miRNAs related to cancer based on miRNA-mRNA regulatory data. First, for genes with abnormal expression that are regulated by more than one miRNA, it is difficult to discriminate which miRNA contributed to the deregulation of this gene. Second, besides miRNA regulation, other factors such as DNA methylation may also result in abnormal expression of the studied gene. To overcome these problems, we defined a novel out-degree (NOD) index to measure the independent regulatory power of an individual miRNA, *i.e.,* the genes uniquely regulated by one specific miRNA. Based on the observation that miRNAs with greater independent regulatory power were more likely to be cancer biomarkers as described in the Results section, we prioritized candidate PCa miRNA biomarkers according to their NOD values, as calculated from the PCa miRNA regulatory network.

In summary, the number of uniquely regulated genes was first computed as a NOD value for each miRNA in the PCa miRNA regulatory network. These miRNAs were further ranked by their NOD values. The Wilcoxon signed-rank test was then applied to assign a statistical significance value (*p*-value) to each miRNA, which indicated whether the NOD value of an individual miRNA was significantly greater than the median level of all these candidate miRNAs. Herein, the threshold of the *p*-value was set at 0.01. Finally, 39 miRNAs were detected as potential PCa miRNA biomarkers in our study.

### Performance comparison with other computational methods

To evaluate the accuracy of our method, we compared its performance with that of two other computational approaches, the miRNA expression fold-change based on the t-test method [34] and another method based on the cancer miRNA synergism theory [12]. The same numbers of top ranked miRNAs as in our prediction results were extracted from these two methods for comparison. The performance of each computational method was expressed as the percentage of known PCa abnormal miRNAs in their prediction results.

### In vitro q-PCR confirmation of candidate prostate cancer miRNA biomarkers

When normal prostate tissue (NPT) samples are unavailable, benign prostatic hyperplasia (BPH) samples can be used as normal prostate samples for comparison with PCa samples [35,36]. The study group consisted of 25 Han Chinese patients with PCa and 20 Han Chinese individuals with BPH with ages ranging from 60 to 91. The PCa and BPH samples were part of a sample set collected for clinical diagnostic tests at the First Affiliated Hospital of Soochow University (Suzhou, China). No extra samples were collected from the study subjects; therefore, verbal consent was obtained from all participating individuals. The study procedure was approved by the ethics committee of Soochow University. The PCa and BPH tissues were snap-frozen in liquid nitrogen and stored at -80°C. Total RNA was extracted with the TRIzol reagent (Invitrogen, China). RNA quantity was measured on a Nanodrop 1000 Spectrophotometer (Thermo Scientific, China). Universal reverse transcription of all the mature miRNAs was performed by enzymatic tailing of the miRNAs by using Poly(A) Polymerase. MiRNAs were first tailed and then reverse transcribed by using universal primers. The sequences of miRNAs were obtained from the miRNAMap database [37]. MiRNA specific primers were designed with Primer 3 software. Quantitative PCR was performed in a volume of 20 μl containing 2 μl of cDNA diluted 10 times, 10 μl of LightCycler® 480 SYBR Green I Master (Roche, China), and 200 nM of each primer. U6 expression was used as the internal control, and all quantitative PCR values were normalized to those of U6 RNA. Triplicates were performed for all reactions with a LightCycler® 480 System (Roche, China). Relative expression was analyzed by the Pfaffl method. All the statistical analyses were carried out on Graphpad Prism software.

### Systematic analysis of the target genes of candidate prostate cancer miRNA biomarkers

The uniquely regulated genes associated with our prediction miRNAs from the PCa miRNA-mRNA target network were retrieved. Gene Ontology (GO) analysis and pathway analysis were performed to explore the relationships between these genes and PCa. The Database for Annotation, Visualization and Integrated Discovery (DAVID) [38] was used for GO annotation and KEGG pathway [39,40] analysis. Another pathway source, MetaCore™ Database from GeneGo Inc., was used for GeneGo pathway mapping analysis. The highly significantly mapped pathways (*p*-value < 0.01) were further confirmed for their association with PCa via NCBI PubMed literature exploration.

## Results

### Global features of the miRNA-mRNA target network

**Figure 2:**
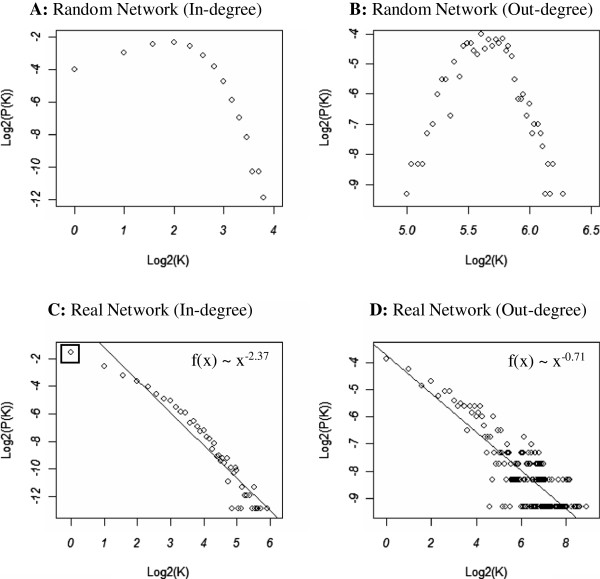
**Degree distribution of the whole human miRNA-mRNA target network.** Panels **(A)** and **(B)** respectively illustrate the In- and Out- degree distributions of the random network, whichwas simulated with the same number of miRNA and mRNA nodes and miRNA-mRNA target pairs. The x-axis represents log-2 based degrees, and the y-axis indicates the log-2 based frequencies of nodes with corresponding degrees. The in-degree and out-degree distributions for the random network were symmetrical while the panels **(C)** and **(D)** show the power law distributions with slopes of -2.37 and -0.71 for the in-degree and out-degree distributions of the real network, respectively. The circle inside the small pane in panel **(C)** represents genes (34.41% of total genes) that were uniquely targeted by an individual miRNA.

Individual miRNAs can regulate multiple genes, and an individual target gene can be co-regulated by several miRNAs [41,42]. However, further exploration of the in-degree distribution of targets and out-degree distribution of miRNAs for the whole human miRNA-mRNA target network revealed that the multiple-to-multiple relationship between miRNA and mRNA is over-emphasized. Compared with the random network simulated with the same nodes and binding links, the in-degree distribution and out-degree distribution of the real miRNA-mRNA target network followed the power-law distribution (see Figure 2), with slopes of -2.37 and -0.71, respectively. This indicated that the distribution of the miRNA regulatory network was also scale-free and similar to other biological complex networks such as the protein interaction network [43].

**Figure 3:**
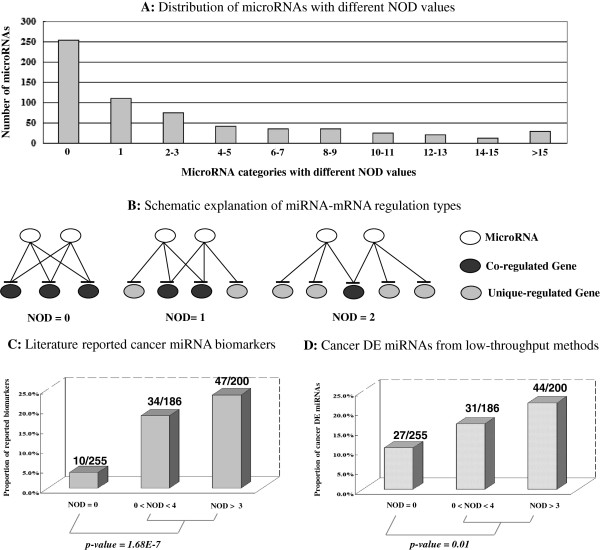
**Schematic explanations of miRNA independent regulatory power and status from cancer miRNA studies.** Panel **(A)** shows the miRNA NOD distribution in the whole human miRNA-mRNA target network. Schematic examples of miRNAs with different NODs are provided in panel **(B)**. The status of literature reported cancer miRNA biomarkers and differentially expressed (DE) miRNAs by low-throughput methods are shown in panel **(C)** and panel **(D)**. The numbers used to compute the percentage in each miRNA group are provided above the corresponding bars (e.g., 10/255 denotes that 10 out of 255 miRNAs within the first miRNA group with a NOD value of 0 were reported as cancer biomarkers in previous studies). The statistical significance levels were computed with Pearson’s chi-square test.

The analysis of our reconstructed miRNA-mRNA network indicated that a large fraction (34.41%) of genes was exclusively regulated by an individual miRNA. Based on this observation, we defined a new index designated as novel out degree (NOD) to measure the independent regulatory power of an individual miRNA (see Figure 3 and Methods). Interestingly, the distribution of miRNA NOD values was also shown to follow the power law. According to their NOD levels, we further classified these miRNAs into three categories: miRNAs with no independent regulatory power (NOD = 0); miRNAs with a small independent regulatory power (0 < NOD < 4); and miRNAs with a large independent regulatory power (NOD > 3). We then explored the current cancer related miRNAs by text mining of PubMed citations. Briefly, we first checked whether each miRNA had been previously claimed as a cancer biomarker by using the NCBI PubMed search engine. Then, to strengthen our findings, we used a previously described method [44] to further explore differentially expressed (DE) miRNAs in cancer detected by low-throughput technology in studies published before January 1^st^ 2013. A statistically significant difference of their biomarker potentials could be observed between miRNAs without independent regulatory power and those with independent regulatory power. The results shown in Figure 3C and Figure 3D indicated that miRNAs with larger independent regulatory power were more likely to be potential cancer miRNA biomarkers and show aberrant functions in cancer.

### Prediction of candidate prostate cancer miRNA biomarkers

Based on the above observation that miRNAs with larger independent regulatory power are more likely to be cancer abnormal miRNAs, we developed a pipeline to infer candidate cancer miRNA biomarkers from cancer conditional miRNA regulatory networks, and then applied this pipeline for PCa study, as described in the Methods section. A total of 39 miRNAs were predicted to be candidate PCa miRNA biomarkers in our study. Among these miRNAs, 20 (51.3%) had previously been shown to be PCa aberrant miRNAs by low-throughput methods in early studies, and thus could be potential miRNA biomarkers in PCa. Among the remaining 19 candidates, 16 miRNAs had been reported to show outlier activities in other cancers, whereas the activities of the other three miRNAs had not been explored yet. These miRNAs were considered potential PCa miRNAs requiring further investigation. Detailed information about the prediction miRNAs and known PCa miRNAs used in this study can be found in Additional files 3 and 4, respectively.

**Figure 4:**
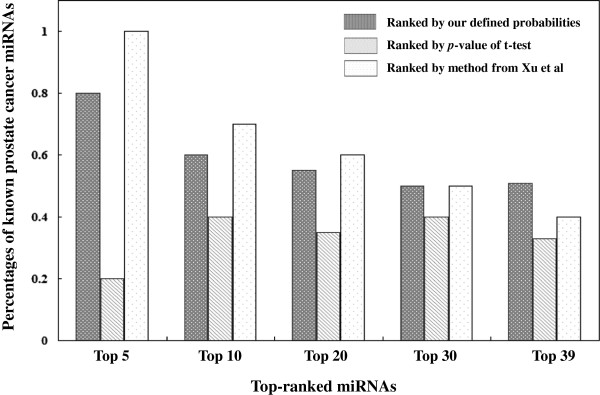
**Performance comparison of the prediction of prostate cancer miRNAs.** This figure summarizes the performance comparison of three PCa miRNA prediction methods – miRNA expression fold-change obtained by the t-test based method, the cancer miRNA synergism theory based method, and our integrative probability based method. The y-axis represents the percentage of known PCa abnormal miRNAs in the top ranked miRNA sets, and the x-axis indicates the top ranked miRNAs from these three approaches.

We further evaluated the performance of our method by comparing our results with data on fold-changes in miRNA expression obtained using the t-test approach [34] and a method based on the cancer miRNA synergism theory [12] (see Methods). The results are shown in Figure 4. As a computational method based on the integration of expression fold-change and miRNA regulatory network feature information, our method performed better than fold-change of t-test based methods. The performance of our method was comparable to that of the method described by Xu et al. [12], based on the reported PCa miRNA information (Figure 4). Nevertheless, Xu’s method is based on a SVM classifier, and is therefore highly dependent on the PCa miRNA training data, whereas our approach does not require any prior knowledge. Overall, these results indicated the satisfactory performance of our prediction method.

### In vitro validation of candidate prostate cancer miRNA biomarkers

**Figure 5:**
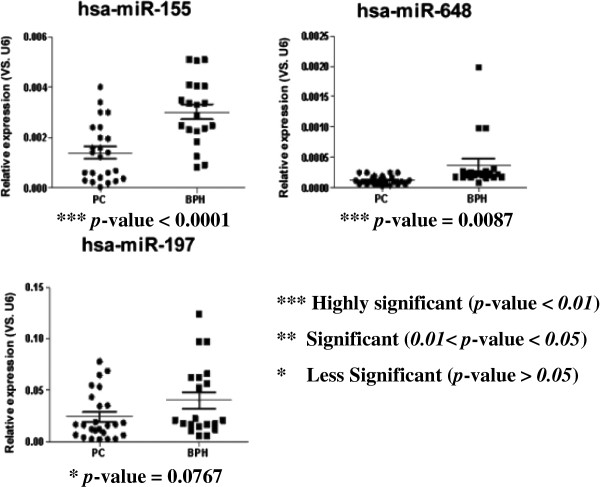
**q-PCR results for selected candidate prostate cancer miRNA biomarkers.** Three miRNAs were randomly selected from our prediction results to detect their outlier activity in PCa samples by using the q-PCR method. Endogenous U6 expression was used as control for normalization. The statistical significance of differences between groups was calculated using the Student’s t-test.

To further investigate the activities of PCa miRNA biomarkers predicted by our approach, we randomly selected three miRNAs (*miR-155*, *miR-648*, and *miR-197*) to detect differences in their expression between PCa tissues and benign prostatic hyperplasia tissues by q-PCR technology. The detection results are shown in Figure 5. Two out of 3 miRNAs (66.7%) were differentially expressed in the two groups of samples (*p*-value < 0.01).

Among these two miRNAs, *miR-155* downregulation in PCa was in agreement with the results of a previous report [45], whereas *miR-648* was identified as a novel PCa miRNA biomarker by our study. Although *miR-197* did not show significant outlier activity in PCa, it was previously proposed as a potential miRNA biomarker for lung cancer in another study [46]. The experimental analysis of the activities of outlier miRNAs verified the reliability of our method.

### Functional analysis of target genes of candidate prostate cancer miRNA biomarkers

In our study, the predicted candidate PCa miRNA biomarkers, along with their uniquely regulated genes (see Additional file 3), provide potential miRNA-mRNA target pairs in PCa. The unique target genes regulated by these candidate miRNAs may also be involved in PCa, assuming that the predicted miRNAs are true PCa miRNAs. To validate our hypothesis, we retrieved the uniquely regulated genes of our predicted miRNAs from the PCa miRNA-mRNA target network, and explored their relationships with PCa through GO analysis and Pathway enrichment analysis (see Methods).

**Figure 6:**
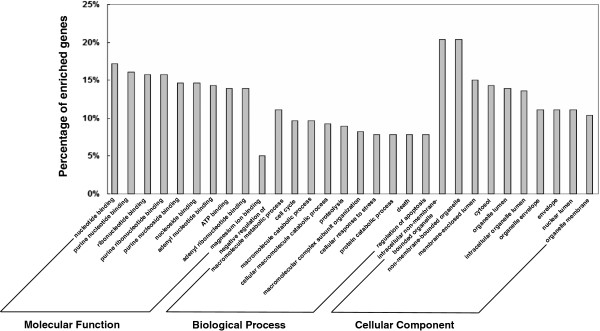
**Gene ontology annotation for the uniquely regulated genes of the predicted prostate cancer miRNA biomarkers.** The uniquely regulated genes of the predicted PCa miRNA biomarkers identified by our method were retrieved and annotated with DAVID tools in three domains of gene ontology: Molecular Function, Biological Process, and Cellular Component. The top 10 significantly enriched items for each domain are shown.

The Database for Annotation, Visualization, and Integrated Discovery (DAVID) [38] was applied for the Gene ontology (GO) annotation at three levels: molecular function, biological process, and cellular component. The ten most highly enriched items for each domain are shown in Figure 6. The results indicated that these genes are well mapped in several PCa associated biological processes, such as cell cycle [47] and regulation of apoptosis [48]. These results further confirmed the accuracy of the predicted PCa miRNA biomarkers to a certain extent.

**Figure 7:**
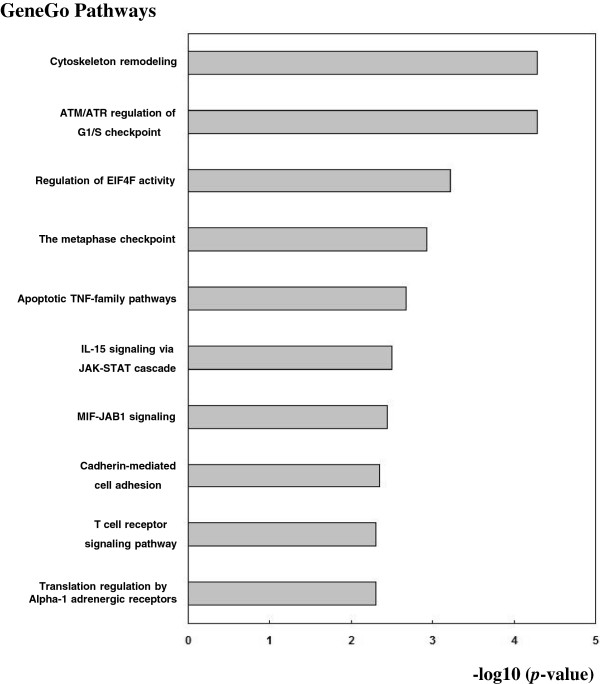
**GeneGo pathway enrichment analysis for uniquely regulated genes of predicted prostate cancer miRNA biomarkers.** The uniquely regulated genes of the predicted PCa miRNA biomarkers from our method were retrieved and mapped in the GeneGo Database. The statistical significance level (*p*-value) was negative 10-based log transformed. The top 10 significantly enriched pathways are shown.

We used the DAVID and the MetaCore™ Database from GeneGo Inc. to map these outlier genes in KEGG pathways and MetaCore™ pathways, respectively. Statistically significantly enriched pathways (p-value < 0.01) from these two datasets were retrieved. For KEGG pathways, we found that these target genes were only considerably enriched in one PCa related pathway termed as “Pathways in cancer”. The top 10 highly enriched pathways from the GeneGo Database were plotted in Figure 7. Significantly enriched pathways, such as cytoskeleton remodeling [49], have been previously shown to be PCa related pathways. Text mining searches in NCBI PubMed were used to explore the relationships between enriched pathways and PCa. The results showed that 20 out of 24 (87.5%) pathways found in the GeneGo database were related to PCa. The PubMed citations regarding the association of these pathways with PCa are listed in Additional file 5. We also compared our enrichment pathways with novel PCa associated pathways detected using other systematic methods [50]. Of 24 enriched pathways from the GeneGo database in our study, 18 (75%) were common pathways with those of previous studies. Taken together, these analyses confirmed the correlation between the target genes and PCa, and thus verified the reliability of our predicted PCa miRNAs.

## Discussion

Previous studies have provided evidence of multiple-to-multiple relationships between miRNAs and their target genes [41,42,51]. From the average view of the miRNA-mRNA target network, that conclusion seems reasonable. Indeed, there are on average 51 target genes for each individual miRNA, and 4 co-regulator miRNAs for each gene in the whole miRNA-mRNA targeting network according to our analysis of the reconstructed network, as described in the Methods section. Based on this theory, numerous attempts have been made to predict cancer related miRNA regulatory modules [14], and cancer miRNAs were shown to have more synergism with their co-regulatory effects on the same genes [18].

**Figure 8:**
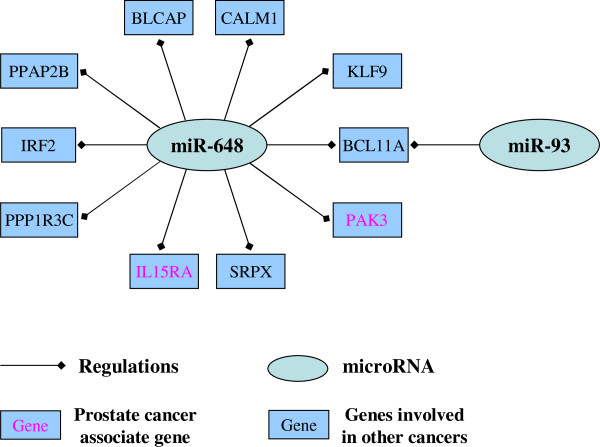
**Target genes of novel identified biomarker -*miR-648*(NOD = 9).** The target genes of query miRNA were retrieved from above collected prostate cancer miRNA-mRNA binding network. Genes that were reported to associate with prostate cancer progress were marked in red.

In the present study, we conducted an in-depth exploration of this matter. Our results revealed the scale-free feature of the miRNA-mRNA target network. We introduced the NOD index to measure the independent regulatory power of an individual miRNA. Contrary to the functional synergism of cancer miRNAs, our data showed that miRNAs with greater independent regulatory power were more likely to be potential biomarkers in human cancers. Based on this evidence, we developed a novel integrative method to infer candidate cancer miRNA biomarkers from the miRNA regulatory network by linking paired miRNA and gene expression data, and highly reliable miRNA-mRNA target data. This pipeline was further applied to PCa. A total of 39 miRNAs were predicted as potential PCa miRNA signatures. Among these miRNAs, 20 have previously been reported to be PCa aberrant miRNAs by low-throughput methods, and 16 miRNAs were shown to be deregulated in other cancers. In vitro q-PCR experiments and functional analysis further verified the accuracy of our predictions and miR-648 was identified as a novel candidate PCa miRNA biomarker. The target genes of miR-648 are listed in Figure 8. With the exception of BCL11A, the other nine genes are exclusively regulated by miR-648. Among these 10 genes, PAK3 and IL15RA were previously shown to be involved in PCa progression [52,53], and PAK3 was identified as an epigenetic biomarker for the prognostic diagnosis of PCa [52]. Despite the fact that no reports on the association of the remaining genes with PCa were found, these genes were shown to be associated with the progression of other cancers, such as breast cancer [54] pancreatic cancer [55] and leukemia [56]. These evidences support that miR-648 could be involved in PCa and act as a novel molecular biomarker for PCa diagnosis.

The miRNA-mRNA network reconstructed in this study consisted of experimentally validated data and computational predicted data. The data resources of the computational prediction databases used in this study, HOCTAR [25], ExprTargetDB [26], and starBase [27], were derived from the predictions based on gene expression information, such as microarray data and Next-generation sequencing data. Therefore, this predicted data should be more accurate than data predicted by programs merely based on sequence level, such as TargetScan [57] and RNAhybrid [58]. The reliable miRNA-mRNA targeting data could guarantee the accuracy of the predicted activities of outlier miRNAs.

The present results provide a basis for the development of algorithms for cancer miRNA biomarker identification. Indeed, two points require further improvement. Firstly, as the gene transcriptional expression data do not reflect changes in protein expression levels [11], the cancer miRNA activity cannot be predicted by our method for miRNAs that function through translational repression. Secondly, the detailed outlier patterns (up-regulation or down-regulation) for the prediction of outlier miRNAs need to be further explored. The integration of protein expression data, transcription factor (TF) information and other omics data is a potential method to improve the prediction. This information will be incorporated in future studies aimed at further developing and refining our method.

## Conclusions

The present analysis revealed novel distinctive features of cancer miRNA biomarkers. A novel bioinformatics framework was proposed to infer candidate cancer miRNA biomarkers from a miRNA regulatory network. The methodology may accelerate the discovery of novel miRNA signatures for cancer diagnosis and treatment, and should also be feasible for the study of other diseases.

## Competing interests

The authors declare they have no competing interests.

## Authors’ contributions

WZ designed the miRNA biomarker prediction pipeline, performed the statistical analysis and drafted the manuscript. FG and JZ carried out the in vitro validation experiments. XJ performed the data collection process. WY, ZS and DY participated in the functional enrichment analysis. BS conceived and coordinated the overall study and revised the manuscript. All authors read and approved the final manuscript.

## Supplementary Material

Additional file 1 **MicroRNA and gene expression datasets.** Paired miRNA and gene expression profiles from the same samples of four prostate cancer and four benign prostate hyperplasia individuals were used. Detailed information about these datasets is provided in this table. Click here for file

Additional file 2 **Comparison of the prediction prostate cancer miRNA biomarkers with different outlier miRNA (gene) thresholds.** MicroRNAs with high statistical significance *(p-*value *< 0.01, Wilcoxon signed-rank test*) were considered as candidate prostate cancer miRNA biomarkers. MiRNAs common to three groups are highlighted in yellow, while miRNAs common to two groups are marked in red. Click here for file

Additional file 3 **Candidate prostate cancer miRNA biomarkers predicted from our method.** MicroRNAs with high statistical significance *(p-*value *< 0.01, Wilcoxon signed-rank test*) were considered as candidate prostate cancer miRNA biomarkers. The references on abnormally expressed miRNAs previously validated in prostate cancer or in other cancers are listed in this table. Click here for file

Additional file 4 **Aberrantly expressed miRNAs in prostate cancer detected by low-throughput methods.** Detailed information about known aberrantly expressed miRNAs in prostate cancer was extracted from previous publications through text mining with the NCBI pubmed engine. Click here for file

Additional file 5 **Significantly enriched GeneGo pathways for the uniquely regulated genes of candidate prostate cancer miRNA biomarkers.** MetaCore™ was used for GeneGo pathway enrichment for the uniquely regulated genes of our prediction prostate cancer miRNA biomarkers. The significant enrichment pathways (*p-*value < 0.01) and the citations reporting their association with prostate cancer are listed in this table. Common pathways with those identified by Wang et al [50] are marked in yellow. The PubMed citation count may change with the updating of the database. Click here for file
